# The Implications of SARS-CoV-2 Infection in a Series of Neuro-Ophthalmological Manifestations—Case Series and Literature Review

**DOI:** 10.3390/jcm12113795

**Published:** 2023-05-31

**Authors:** Nicoleta Anton, Camelia Margareta Bogdănici, Daniel Constantin Brănișteanu, Ovidiu-Dumitru Ilie, Irina Andreea Pavel, Bogdan Doroftei

**Affiliations:** 1 Surgery Department, Faculty of Medicine, University of Medicine and Pharmacy, 700115 Iasi, Romania; dbranisteanu@yahoo.com (D.C.B.); irinaandreea.pavel@gmail.com (I.A.P.); bogdandoroftei@gmail.com (B.D.); 2 Ophthalmology Clinic, St. Spiridon County Emergency Hospital, 700111 Iasi, Romania; 3 Department of Biology, Faculty of Biology, Alexandru Ioan Cuza University, 700505 Iasi, Romania; ovidiuilie90@yahoo.com

**Keywords:** optic neuritis, orbital cellulitis, anterior ischemic optic neuropathy, venous thrombosis, ocular pathology, SARS-CoV-2, ocular manifestations in COVID infections

## Abstract

The global pandemic impact of the COVID-19 infection included clinical manifestations that affected several organs and systems, with various neuro-ophthalmological manifestations associated with the infection. These are rare and occur either secondary to the presence of the virus or by an autoimmune mechanism secondary to viral antigens. The manifestations are atypical, being present even in the absence of the systemic symptoms typical of a SARS-CoV-2 infection. In this article, we introduce a series of three clinical cases with neuro-ophthalmological manifestations associated with COVID infection that were shown in Ophthalmology Clinic of St. Spiridon Emergency Hospital. Case 1 is that of a 45-year-old male patient with no personal history of general pathology or ophthalmology, with binocular diplopia, painful red eyes, and lacrimal hypersecretion with a sudden onset of about 4 days. Based on the evaluations, a positive diagnosis of orbital cellulitis in both eyes is made. Case 2 is that of a 52-year-old female patient with general PPA (personal pathological antecedents) of SARS-CoV-2 infection 1 month prior to presentation with decreased visual acuity in the right eye and a positive central scotoma, preceded by photopsia and vertigo with balance disorders. The diagnosis is made at the right eye for retrobulbar optic neuritis and post-SARS-CoV-2 infection status. The last clinical case is that of a 55-year-old male patient known to have high blood pressure (HBP) with a sudden, painless decrease in VARE approximately 3 weeks post-SARS-CoV-2 immunization (Pfizer vaccine first dose). The diagnosis is made after consulting all the RE results for central retinal vein thrombosis. Conclusions: Although the cases were quickly and efficiently investigated and the treatment was administered adequately by a multidisciplinary team (cases 1 and 3), the evolution was not favorable in all three situations. Atypical neuro-ophthalmological manifestations can also be present in the absence of systemic symptoms typical of SARS-CoV-2 infection.

## 1. Introduction

The emergence of COVID-19 at the end of 2019 determined a particular series of cases that, in addition to respiratory manifestations, also had neurological manifestations, the virus being able to produce a wide range of systemic manifestations. In a 2022 review regarding the neurological changes associated with COVID infection in case series and group studies, Matthew K. et al. reported neurological manifestations in 14–57% of hospitalized patients with COVID-19, with taste and smell disturbances reported in many patients with milder disease. In this study, the incidence of ocular manifestations among patients with COVID-19 varies between groups, with an estimated incidence of up to 30% in hospitalized patients [1]. Presentations range from completely asymptomatic carriers to multiple organ failure and death. The virus can affect the eye in several ways, and there is a wide range of ocular manifestations, with symptoms including irritation (chemosis), excessive lacrimation (epiphora), and ocular secretions showing conjunctivitis up to severe types with cranial nerve damage and neuro-ophthalmological manifestations. In a series of 38 hospitalized patients with COVID-19 in China, 12 (31.6%) patients had ocular symptoms before systemic symptoms [1,2,3,4,5,6].

Cases of patients who, within the context of the COVID-19 infection or secondary to immunization, were diagnosed with optic neuritis, cranial neuropathies, or optic neuromyelitis are cited in the literature. They have been reported in various studies as dysfunctions of the optic nerve, movement anomalies of the eyeballs, and visual field defects. Studies show that these neuro-ophthalmological manifestations can occur in isolation or in association with neurological syndromes. Manifestations include headache, eye pain, visual disturbances, diplopia, cranial nerve palsies secondary to Miller-Fisher syndrome, Guillain-Barré syndrome, or encephalitis, and nystagmus. The authors consider that some of the ocular and neuro-ophthalmological changes were not detected in time due to the general severity of the COVID-19 virus infection, which did not allow a complete ophthalmic evaluation in the anesthesia and intensive care departments [1,2].

As related to vaccination against the coronavirus disease 2019 (COVID-19), this was implemented to eliminate the SARS-CoV-2 pandemic, providing significant protection against infection and the development of multi-organ acute respiratory distress syndrome. However, subsequent studies have demonstrated potential adverse events in vaccinated patients, including ocular complications: non-arteritic anterior ischemic optic neuropathy (NAION), central serous chorioretinopathy (CSC), and Vogt-Koyanagi-Harada (VKH) disease have been reported [2,3,4,5,7].

Regarding these cases cited in the literature that occur after the COVID immunization, they are not highly numerous. Several mechanisms that are associated with secondary ophthalmological manifestations have been reported: the receptor for angiotensin 2, which is found in the central nervous system but also inside the retinal vessels [5], through a direct mechanism that leads to the dysfunction of endothelial cells, causing ischemia and coagulopathy [5,8,9,10].

Likewise, other studies claim that the neurological manifestations occur secondary to viremia crossing the blood-brain barrier or through infected leukocytes [5,9,10,11].

This study reports a series of three clinical cases of patients with neuro-ophthalmic diseases that occurred within the context of the COVID-19 pandemic and immunization against COVID.

## 2. Presentation of Cases

### General Information about Patients

We introduce a series of three patients who presented themselves in the Ophthalmology Clinic of St. Spiridon Hospital with neuro-ophthalmic manifestations associated with the COVID infection in 2021, when vaccination was also possible: a 45-year-old male patient with bilateral orbital cellulitis, which occurred as a result of the SARS-CoV-2 infection; a 52-year-old female patient with post-SARS-CoV-2 optic neuritis; and a 55-year-old male patient with secondary ischemic optic neuropathy prior to SARS-CoV-2 immunization. All patients were fully evaluated and signed informed consent in order to carry out all investigations and appropriate treatment. The ophthalmic evaluation consisted of: visual acuity measurement, IOP determination, chromatic sense, visual field determination, ocular motility examination, interdisciplinary examinations, and brain imaging.

**Figure 1:**
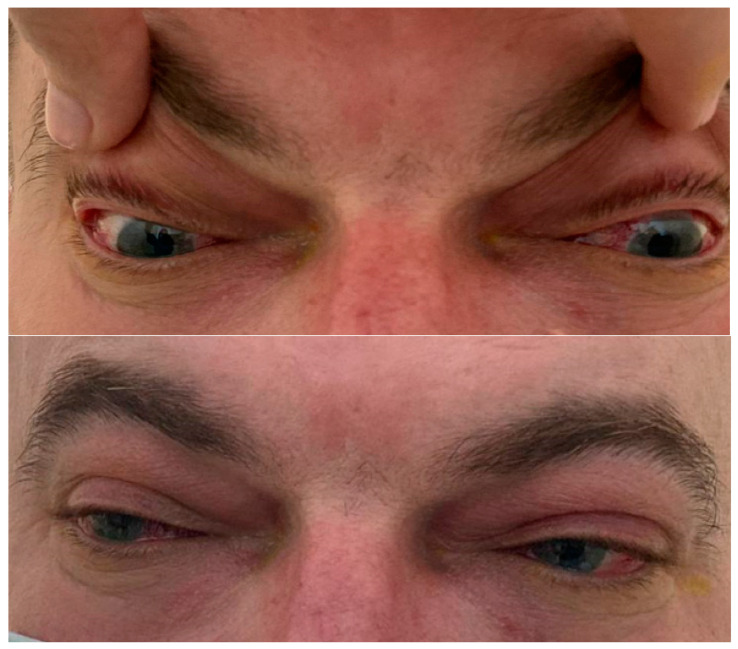

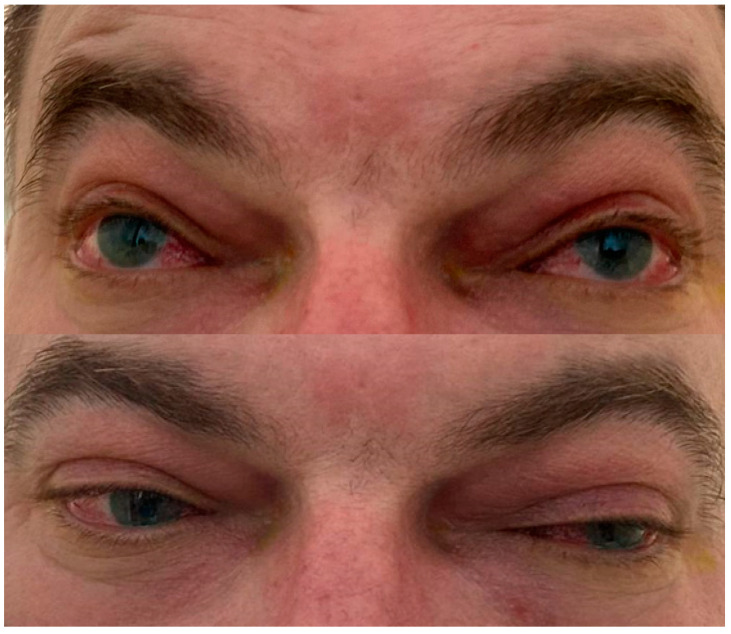
Examination of ocular motility and the segment prior to admission, case 1, slight limitation of abduction (COVID-19 isolation area).

**Figure 2:**
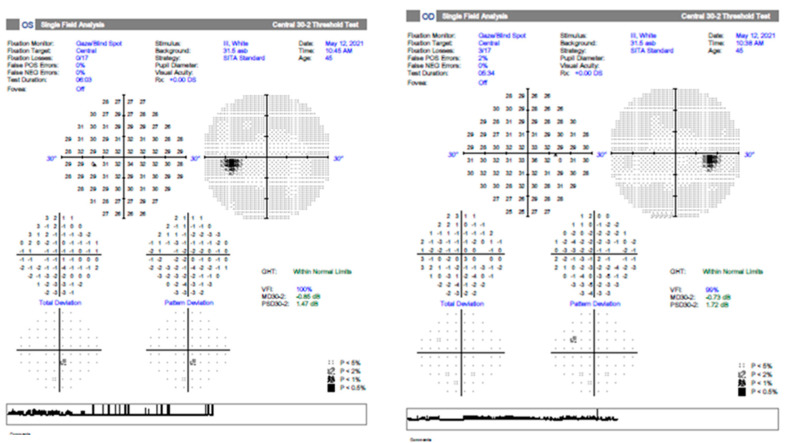

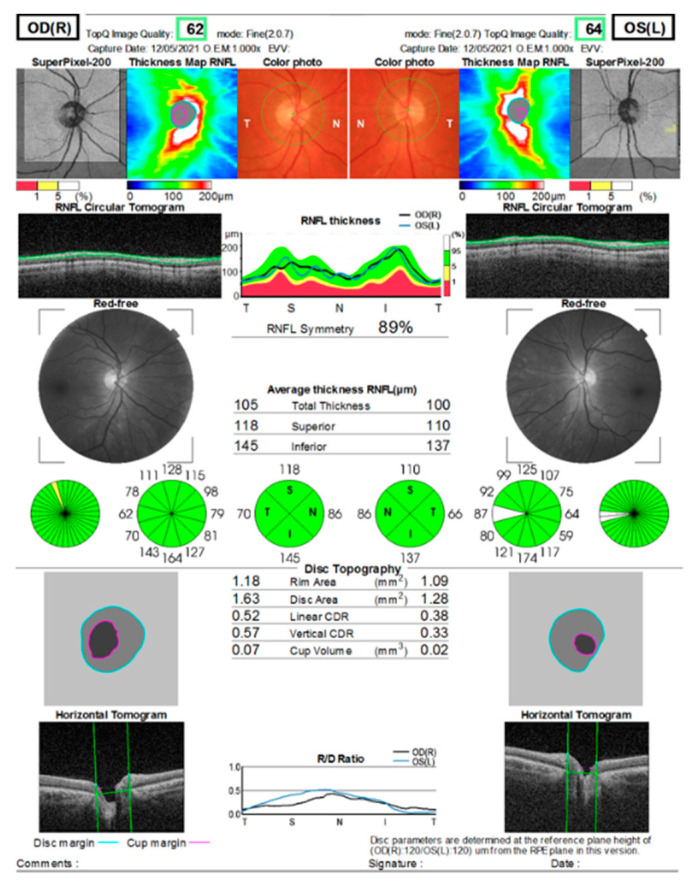
Visual field and OCT examination in both eyes 1 month after the presentation (12 may 2021) of case 1 (not possible immediately due to the COVID infection).

Case 1 is that of a 45-year-old male patient with no personal history of general pathology or ophthalmology who presented with BE binocular diplopia to the eye emergency department, painful red eyes, and lacrimal hypersecretion with a sudden onset of approx. 4 days (Figure 1) in April 2021. The functional ophthalmic examination reveals an VA BE (visual acuity both eyes) of 0.4 with correction cc (−2.75 sf) and an IOP (intraocular pressure) RE of 24 mmHg and an IOP LE of 23 mmHg. Biomicroscopic examination of the anterior segment reveals significant conjunctival congestion, dilated episcleral vessels, corneal epithelial edema 2+, and periocular skin congestion. The fundus is normal. Ocular motility: BE (both eyes) slight limitation of abduction. According to the protocol, the patient tested positive for SARS-CoV-2 virus infection. We mention the fact that the patient was asymptomatic while being isolated in a special room. Blood tests reveal leukocytosis with neutrophilia, thrombocytosis, and an inflammatory syndrome with elevated ESR and CRP; the OCT examination reveals a normal visual field without systematic changes or thinning of the retinal nerve fiber layers (Figure 2). The tests for HBV, HCV, HIV, TPHA, FT4, and TSH were negative. Craniocerebral computer tomography (CT) with contrast material diagnoses bilateral orbital cellulitis with infiltrating appearance of intra- and extraconal fat, infiltration of bilateral malar and palpebral subcutaneous cellular tissue, and discreet infiltrative appearance of the internal rectus muscles (RE 4.7 mm, LE 5.4 mm). The examination of infectious diseases, otolaryngology (ENT), and maxillofacial surgery (MFS) does not identify etiologies responsible for the clinical picture. Based on the evaluations, a positive diagnosis of BE orbital cellulitis is made. The evolution is slowly favorable, with gradual remission of clinical signs and recovery of visual acuity under treatment with Ceftriaxone (generally 2 g/day), Vancomycin, Aerius (desloratadine 5 mg), Moxifloxacin, Betabioptal (betamethasone 0.2 g), chloramphenicol 0.5 g, and Naabak (Sodium isospaglumate) (topical administration, initially six times a day). Thus, after 1 month, an improvement in eye movements, a reduction in periocular inflammation, a normal visual field, an OCT examination, and an improvement in visual acuity can be seen, indicating a favorable prognosis.

**Figure 3:**
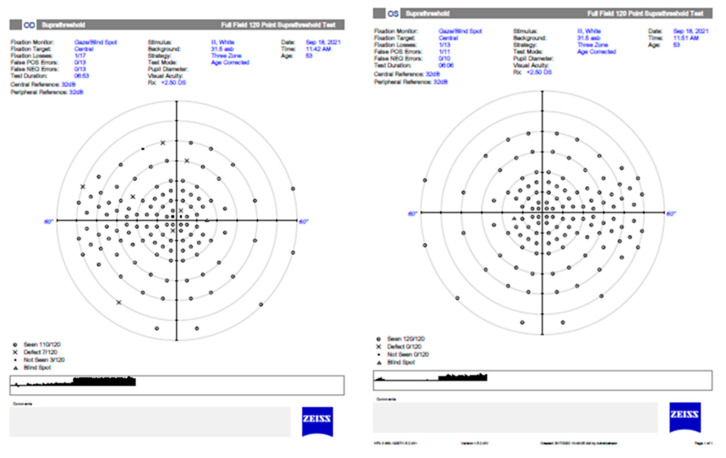
The RE visual field with a centrocecal scotoma at hospitalization (Full Field 120 points Suprathreshold). LE normal (case 2).

**Figure 4:**
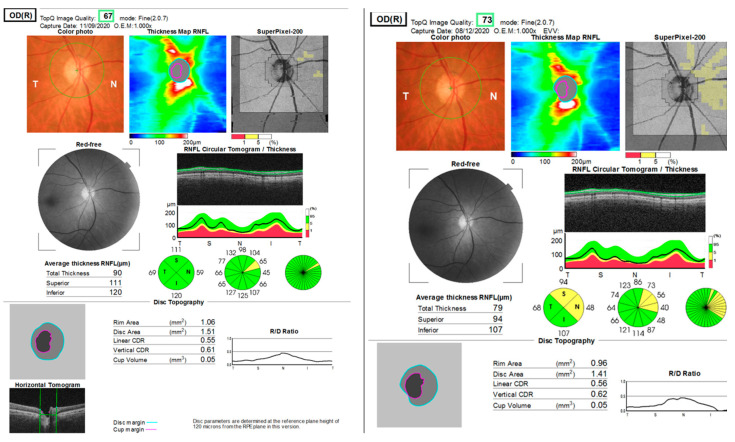
OCT at hospitalization (11 September 2020) vs. 3 months after treatment (8 December 2020) (thin RE superior and nasal SNFR, 136 vs. 94 and 66 vs. 48 µm) if there is also a visual field at discharge (case 2).

Case 2 is that of a 52-year-old female patient with general APP of SARS-CoV-2 infection 1 month prior to presentation in September 2021. She complained of decreased VARE and a positive central scotoma, preceded by photopsia and vertigo with balance disorders. After the functional examination revealed the VA RE is 1, VA LE is 1.2, IOP RE is 13 mmHg, and IOP LE is 12 mmHg, the anterior segment examination is within normal limits, without Relative Afferent Pupillary Defect (RAPD), and color sense and ocular motility are normal. The OF RE (ocular fundus) optic nerve papilla has an erased contour in the nasal sector. Blood tests are within normal limits: negative HIV, negative RPR, normal rheumatoid factor, liver and kidney samples, HLG, and normal coagulation. A contrast-enhanced MRI is normal. VF RE (visual field right eye) centrocecal scotoma (Figure 3) and normal LE (left eye); OCT macula and optic nerve normal; X-ray chest aortic atheromatous; normal bilateral X-ray of sacroiliac joints. After gathering the data, the diagnosis is made at the RE (right eye) of retrobulbar optic neuritis and post-SARS-CoV-2 infection status. The therapy is initiated with metilprednisolon iv (intravenous route) for 3 days at 1 g (1000 mg) per day and then decreased to 1 mg/kg/body for 11 days. Under treatment, the general condition of the patient gradually recovered with the gradual remission of changes in the visual field. At the 3-month evaluation, the optic nerve OCT examination reveals the sequelae of optic neuritis, namely a moderate thinning of the retinal nerve fiber layers in the superior and nasal sectors without affecting the visual acuity (Figure 4).

**Figure 5:**
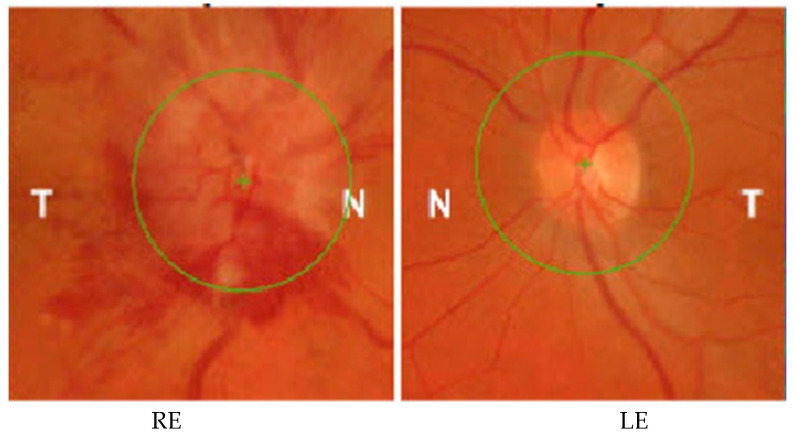
The optic nerve during the fundus examination with papillary edema revealed in the RE, normal aspect in the LE.

Case 3 is that of a 55-year-old male patient known to suffer from hypertension. He suffers from a sudden, painless decrease in VA RE approximately 3 weeks post-SARS-CoV-2 immunization (Pfizer vaccine first dose). VA RE PL uncertain, VA LE 0.7 fc, 1 corrected, IOP BE with normal values. Biomicroscopic examination of the LE anterior pole for conjunctival melanosis, crystalline transparency disorders, and otherwise normal. OF RE papillary edema, peripapillary hemorrhages in the flame, dilated veins due to the emergency, and at normal LE of the optic nerve (Figure 5).

**Figure 6:**
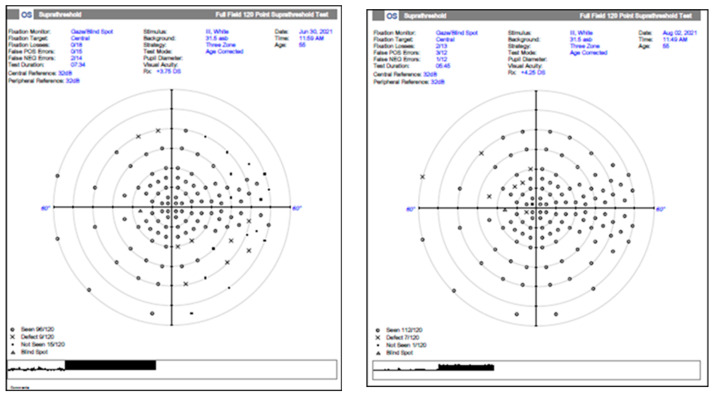
The visual field (Full Field 120 points Suprathreshold test) of the LE upon hospitalization (June 2021) and after 2 months (August 2021), relative scotoma without systematization.

**Figure 7:**
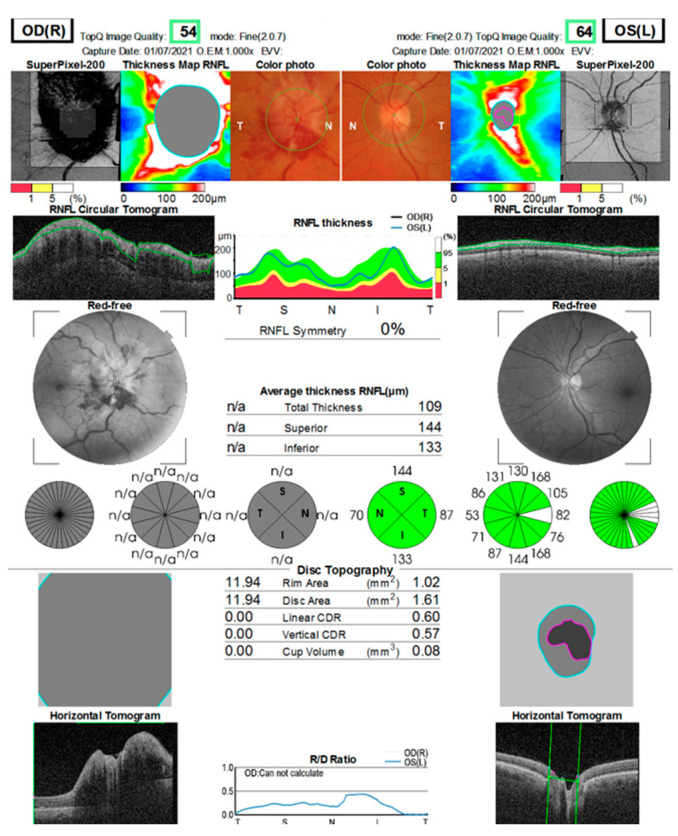
OCT of the optic nerve. RE diffusely thickens SNFR in all sectors (papilar edema); LE normal.

Blood tests revealed mild hypercholesterolemia and inflammatory markers within normal limits. ANA, RPR, and Ac anti-HIV tests within normal limits (antinuclear antibodies, rapid plasma reagin test for treponema pallidum, and acquired immune deficiency syndrome). The inter-clinical examinations of cardiology, dermatology, ENT (otorhinolaryngology), maxillofacial surgery (MFS), hematology, and neurology did not raise the suspicion of another etiology. Contrast-enhanced MRI examination: right optic nerve 10 mm with moderate contrast uptake. Normal chest X-ray. The visual field (Full Field 120 points Suprathreshold test) of the LE upon hospitalization (June 2021) and after 2 months (August 2021) shows relative scotoma without systematization (Figure 6). The OCT examination reveals a significant diffuse thickening of the optic nerve and a normal aspect of the LE. A normal visual field of the LE, while this is not possible due to poor visual acuity in the RE (Figure 7).

Furthermore, in order to establish the etiology, thus preventing an episodic event in the left eye, the patient is directed again to the laboratory to perform the thrombophilic profile, which reveals the presence of factor XIII, homozygous mutant PAI gene, heterozygous mutant factor V, heterozygous mutant MTHFR C677T, MTHFR A1298C homozygous mutant, and homocysteine with values above 21.2 µmol/mL (ideally <10 µmol/mL). The hematological examination confirmed the diagnosis of hereditary thrombophilia and hyperhomocysteinemia with a recommendation of Clexane (enoxaparin sodium) 0.4 × 2/day (anticoagulant), acetylsalicylic acid (75 mg/day) 1 pc/day, and natalvit (vitamin complex) 1 pc/day for 30 days and subsequent reevaluation. Thus, the diagnosis is made after consulting all the RE results of central retinal vein thrombosis within the context of the COVID-19 infection, with hereditary thrombophilia and hypermocysteinemia as aggravating factors. Under general treatment with Metilprednisolon, Pentoxifylline, Ceftriaxone, enoxaparin sodium 0.4 × 2, aspenter 1 pc per day, and natalvit, no favorable result is obtained, with the visual acuity evolving towards no light perception (NLP).

## 3. Results and Discussions

In the present study, we report two cases that were associated with COVID-19 infection (one case of orbital cellulitis and one of retrobulbar optic neuritis) and one case that was registered after vaccination against COVID-19 with central vein thrombosis of the retina, which had presented a favorable evolution except for the last case, which evolved towards the loss of visual function in the respective eye.

**Figure 8:**
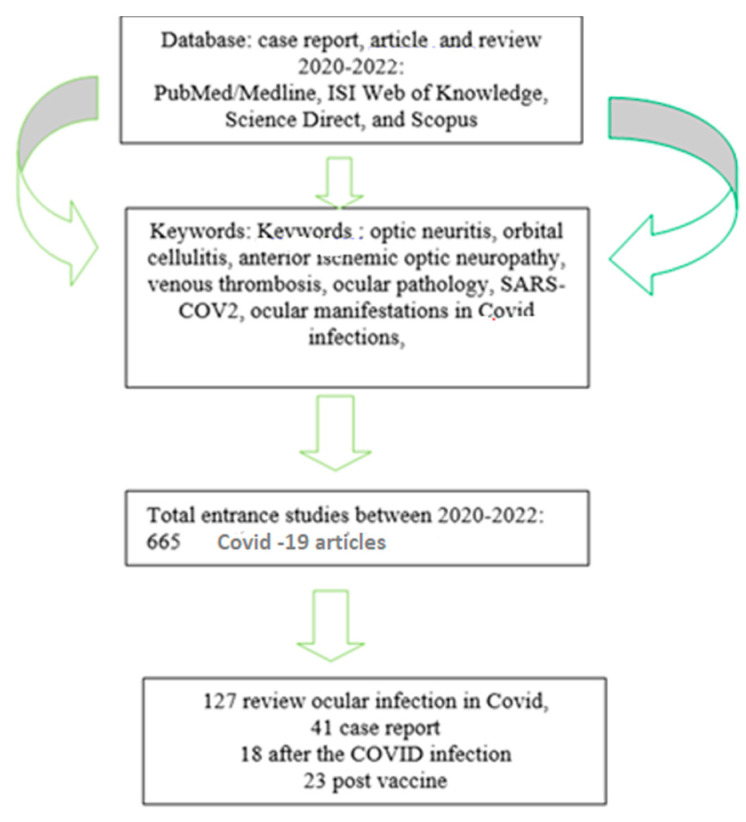
A flowchart of the current survey design, strategy, results, and current studies selected according to the selection criteria.

Coronavirus disease (COVID)-19 is caused by the novel severe acute respiratory syndrome coronavirus 2 (SARS-CoV-2) (as described by the Coronaviridae Study Group of the International Committee on Taxonomy of Viruses 2020) and has a predominant clinical presentation with respiratory disease [6]. Up to 21% of patients with COVID-19 may have systemic neurological signs, and 0.4% have cranial nerve involvement. Potential neuro-ophthalmic associations with COVID-19 include optic neuritis and myelitis, ischemic optic neuropathy, Guillain-Barre syndrome, Miller-Fisher syndrome, and cranial neuropathies such as cranial nerve III or VI palsies [6,9,10,11,12,13,14]. Most studies present cases with an association of ocular manifestations, representing 6–12% of patients with COVID-19. Ocular manifestations may precede systemic symptoms by 3 h to 5 days in 13% of patients [1,13]. In addition to the already known changes at the level of the anterior segment, the studies show the changes that can occur at the level of the posterior eye segment. As happened in our cases, the occlusion of the central vein of the retina and the central artery are two of the several vascular manifestations of COVID-19. Patients with COVID-19 have hypercoagulability, as evidenced by higher levels of fibrinogen, prothrombin time (PT), prothrombin dimer, and activated partial thromboplastin time (aPTT). A literature review showed that COVID-19 was associated with an 8.86-fold risk of retinal vascular microvasculopathy [13]. Another major complication is papillophlebitis, a rare condition characterized by venous congestion and optic disc edema; it manifests as a consequence of inflammation of the retinal veins or, possibly, of the optic capillaries, which occurs in healthy young people and has a benign evolution [14]. Neurological complications reported in studies include optic neuritis and myelitis, ischemic optic neuropathy, Guillain-Barre syndrome, Miller-Fisher syndrome, CNIII or CNVI palsy-like cranial neuropathies, and neuromyelitis optica spectrum disorder. Optic neuritis is an uncommon manifestation of the SARS-CoV-2 infection. One contextual mechanism in which viral antigens would induce an immune response against self-proteins, or direct SARS-CoV-2 infection of the central nervous system, may be involved in the optic nerve damage. A delay in the diagnosis of neuro-ophthalmic manifestations of COVID-19 can lead to irreversible optic atrophy [10,15]. There are few publications in the literature that refer strictly to cases with posterior segment pathology associated with the COVID-19 infection. Between 2020 and 2022 while carrying out a search through the specific research literature by accessing PubMed/Medline, ISI Web of Knowledge, ScienceDirect, and Scopus databases and by using keywords such as: optic neuritis, orbital cellulitis, anterior ischemic optic neuropathy, venous thrombosis, ocular pathology, SARS-CoV-2, ocular manifestations in COVID infections, we identified a number of 665 articles (review, case report, original article) that refer to eye diseases and the COVID infection, of these 127 review articles and open access and 41 case reports with eye pathologies associated with the COVID infection and selected case reports with eye damage after the vaccine, some of which are presented in Figure 8, Table 1 and Table 2.

During the active period of COVID (March 2020–May 2020), when there were restrictions and only urgent cases were admitted, we carried out an analysis in our clinic that included adults. Only 61 of the 616 patients who presented themselves for evaluation were hospitalized, and in most cases, the eye damage was not related to the COVID infection (most frequently, patients were hospitalized with retinal detachment (29.5%), trauma (24.6%), and corneal sclerosis (13.1%), most cases being surgical) [27]. Furthermore, in a similar study that we carried out, including both the non-pandemic period (2016–2019) and the pandemic period (2020), we found out that there were practically no significant differences between the two periods, both in terms of the number of presentations and in the type of pathology, with trauma showing a significant percentage of patients [28]. This can be explained by the fact that, when admitted to the ATI department during the pandemic, most of the patients with serious respiratory damage could not be evaluated from an ophthalmological point of view, making it practically impossible to identify possible eye contact caused by the COVID infection. The cases presented in this case report were identified in 2021, practically 1 year after the top outbreak, in the case of mild types of COVID infection that resulted in severe eye damage in the absence of respiratory symptoms in young people.

From what we have identified as cases, there is no case of bilateral orbital cellulitis recorded in the literature within the context of COVID infection in a young person without general pathological antecedents—a case that had a favorable evolution under treatment with remission of symptoms.

The second case with optic neuritis, with a rather rare association with COVID, as studies say, that usually occurs in multiple sclerosis, brings into discussion that complete multidisciplinary evaluation and imaging play a key role in the diagnostic process. According to other studies, we also recommend that the patient be periodically followed up by both ophthalmologists and neurologists to rule out any other later medical conditions [18,20]. Failing to diagnose the neuro-ophthalmic manifestations of the SARS-CoV-2 infection can lead to treatment delays, additional eye damage, and irreversible vision loss.

Also, the case of central vein thrombosis of the retina within the context of the vaccine in a young person who was unaware of hereditary thrombophilia brings into question the hypothesis that the immune response of the human body to the COVID-19 vaccinations may be involved in the pathogenesis of post-vaccination ocular side effects.

## 4. Conclusions

Neuro-ophthalmological manifestations secondary to contact with the SARS-CoV-2 virus/immunization are rare but must be known. There are three clinical cases with favorable clinical evolution (cases 1 and 2) but also unfavorable within the context of the patient’s presentation at an increased time interval compared to the onset of the symptoms (case 3). Although the cases were quickly and efficiently investigated and the treatment was administered adequately by a multidisciplinary team (cases 1 and 3), the evolution was not favorable in all three situations. Atypical neuro-ophthalmological manifestations may also be present in the absence of systemic symptoms typical of SARS-CoV-2 infection, sometimes preceding respiratory manifestations. An early diagnosis, a multidisciplinary evaluation, and a fast dose of anticoagulant medication when required could have led, especially in case 3, to a favorable evolution and preservation of visual function.

## Figures and Tables

**Table 1:** Summary of the previous case reports (ocular pathology and COVID infections).

Study	Country	Design	Intervention	Population	Result
1. Mohammed A. Azab et al., 2021 [16]	Egypt	Case reports and meta-analysis	One-gram intravenous methylprednisolone for three days, followed by 60 mg oral prednisone, were prescribed for a week with gradual tapering.	A 32-year-old male patient with unilateral optic neuritis, 2 weeks after COVID infection.	The VA improved, but not like before the COVID infection.
2. Jaydeep A. Walinjkar et al., 2020 [17]	India	Report cases	Tree Intravitreal injection of Ranibizumab 0.05 mL of 10 mg/mL concentration with due precautions. The second dose is after 1 month.	A 17-year-old girl with unilateral OVCR 2 days after COVID infections.	Significant improvement in edema and VA after the first doses (from 6/18 to 6/12)
3. Davide Romano et al., 2022 [18]	Italy	Report cases	Intravenous (IV) methylprednisolone 1 g once daily for 5 days.	A 35-year-old female with a positive anamnesis of COVID-19 infection, visual disorders, and neurological symptoms.	Improving symptoms.
4. Rodríguez-Rodríguez M.S. et al., 2021 [10]	Mexico City	Report cases	One g/day of intravenous methylprednisolone for 5 days, followed by an oral prednisone taper.	A 55-year-old woman examined on 25 April 2020, for a 12-day history of headache and unilateral optic neuritis.	At a follow-up visit 3 months later, the ocular pain decreased. Nevertheless, left-eye vision did not improve, so the optical atrophy still manifested despite the treatment.
5. Anuradha Raj et al., 2021 [19]	India	Case report and review	The first intravenous (IV) injection of remdesivir in the dose of 200 mg, followed by daily IV maintenance doses of 100 mg for the next 5 days, azithromycin 500 mg/day IV infusion, and tocilizumab 400 mg IV.	A 37-year-old man, cavernous sinus thrombosis with central retinal artery or secondary to COVID-19 infection occlusion.	Proptosis, ptosis, and ophthalmoplegia recovered completely within 1 month of treatment.
6. Tsu Hong Lim et al., 2021 [20]	China	Report cases	Intravenous (IV) ganciclovir 225 mg twice a day (10 mg/kg/day) for 2 weeks.	A 33-year-old Malay with frosted branch angiitis (FBA) in the right eye 1 month after COVID with acquired immunodeficiency syndrome (AIDS).	It was noted that his FBA in his right eye had improved gradually and that his best corrected visual acuity had recovered to 6/12.
7. Helio F. Shiroma et al., 2022 [21]	Brazil	Report cases	Nine patients received intravitreal injections of anti-angiogenic drugs, and one received ketorolac. Tromethamine drops for the management of secondary macular edema; four were untreated.	Fourteen cases of retinal vascular occlusion within 3 months of laboratory-confirmed COVID-19 infections were identified.	Improving visual acuity and reducing macular edema.

**Table 2:** Summary of the previous case reports (ocular pathology and COVID vaccine).

Study	Country	Design	Intervention	Population	Result
1. Gustavo Savino, et.al, 2022 [22]	Italy	Case report series	Patients were females, 64, 58, and 45 years old, respectively. MRI showed enlargement of all right rectus muscles, with both belly muscle and insertion involvement in the first case associated with right scleritis.	The first and second patients were treated, respectively, with oral and topical glucocorticoids with a complete clinical response. Two cycles of oral non-steroidal anti-inflammatory drugs were administered to the third patient with a partial response.	A rapid clinical improvement after 2–4 days reported improvement of pain and diplopia, but no change in the proptosis
2. Sonia Valsero Franco et al., 2022 [23]	Spain	Case report series	No specific treatment was given.	A 53-year-old man who presented a visual field defect in the right eye 7 days after the first vaccine dose. A 65-year-old man who presented anterior optic disc neuropathy 12 days after his first vaccination.	Case 1: The OCT showed a slight loss of ganglion cells in both eyes, and the RNFL showed atrophy. Case 2: OCT showed atrophy of the temporal quadrants of the RNFL and a general loss of ganglion cells in the affected eye.
3. Wumeng Jin et al., 2021 [24]	China	Report cases	under chronic treatment with methylprednisolone (Medrol) 10 mg/d, mycophenolate mofetil (Cycopin) 1 g/d, tacrolimus (Prograf) 1.5 mg/d, and perindopril (Acertil) 4 mg/d. The patient was symptomatically treated with levofloxacin eye drops on the third day after the symptom onset, when the conjunctival congestion was already significant in the left eye. Diclofenac sodium eye drops and ganciclovir ophthalmic gel were added to the treatment.	A 28-year-old female suffering from SLE for six years. The patient received an inactivated COVID-19 vaccine on 6 April 2021.	Finally, the patient reported that the lesions faded ten days after symptom onset.
4. Rachna Subramony et al., 2021 [25]	California	Report cases	She underwent bilateral vitrectomies for simultaneous rhegmatogenous retinal detachments.	A 22-year-old woman with myopia but no ocular trauma or other major medical history presented to the emergency department with 5 days of progressive, painless vision loss in her right eye.	She had follow-up visits with ophthalmology on postoperative days 1, 6, and 13, with reportedly improved field of vision and acuity in the right eye.
5. Kyohei Tsuda et al., 2019 [26]	Japan	Report cases	No specific treatment was given.	A healthy 18-year-old Japanese female noticed a floater in the left eye 1 day after the second vaccination for coronavirus disease 2019. Fundus examination revealed retinal and optic disc hemorrhage.	The hemorrhage resolved spontaneously within 5 months.
6. Ruyi Han et al., 2022 [7]	China	Case report and review	Systemic prednisone was administered, Complicated CSC may develop in the eyes with vaccine-related VKH after steroid treatment. By gradually replacing prednisone with cyclosporine within 2 months, the subretinal fluid was completely absorbed at the last visit.	A 62-year-old Chinese man who developed Vogt-Koyanagi-Harada (VKH) disease six days after his third dose of an inactivated COVID-19 vaccine, with a preceding severe headache and tinnitus. Another three and a half months later, the visual acuity of his right eye slightly decreased due to complications of the central serous chorioretinopathy (CSC) disease.	Completely relieved inflammation and improved visual acuity.

## Data Availability

The data published in this research are available on request from the first author and corresponding authors.

## References

[1] Matthew, Hensley K., Markantone D., Prescott H.C. (2022). Annual Review of Medicine Neurologic Manifestations and Complications of COVID-19. www.annualreviews.org • Neurologic Manifestations of COVID-19. Annu. Rev. Med..

[2] Luís M.E., Hipólito-Fernandes D., Mota C., Maleita D., Xavier C., Maio T., Cunha J.P., Ferreira J.T. (2020). A Review of Neuro-Ophthalmological Manifestations of Human Coronavirus Infection. Eye Brain.

[3] Betsch D., Paul R. (2021). Neuro-Ophthalmologic Manifestations of Novel Coronavirus. Adv. Ophthalmol. Optom..

[4] Bolletta E., Iannetta D., Mastrofilippo V., De Simone L., Gozzi F., Bonacini M., Belloni L., Zerbini A., Adani C., Salvarani C. (2021). Uveitis and Other Ocular Complications Following COVID-19 Vaccination. J. Clin. Med..

[5] Schrage N., Blomet J., Holzer F., Tromme A., Ectors F., Desmecht D. (2022). Eye Infection with SARS-CoV-2 as a Route to Systemic Immunization?. Viruses.

[6] Leung E.H., Fan J., Flynn H.W., Albini T.A. (2022). Ocular and Systemic Complications of COVID-19: Impact on Patients and Healthcare. Clin. Ophthalmol..

[7] Han R., Xu G., Ding X. (2022). COVID-19 Vaccine-Related Vogt–Koyanagi–Harada Disease Complicated by Central Serous Chorioretinopathy during Treatment Course: Case Report and Literature Review. Vaccines.

[8] Burgos-Blasco B., Güemes-Villahoz N., Donate-Lopez J., Vidal-Villegas B., García-Feijóo J. (2021). Optic nerve analysis in COVID-19 patients. J. Med. Virol..

[9] Sawalha K., Adeodokun S., Kamoga G.-R. (2020). COVID-19-Induced acute bilateral optic neuritis. J. Investig. Med. High. Impact Case Rep..

[10] Rodríguez-Rodríguez M.S., Romero-Castro R.M., Alvarado-de la Barrera C., González-Cannata M.G., García-Morales A.K., Ávila-Ríos S. (2021). Optic neuritis following SARS-CoV-2 infection. J. Neurovirol..

[11] Mabrouki F.Z., Sekhsoukh R., Aziouaz F., Mebrouk Y. (2021). Acute Blindness as a Complication of Severe Acute Respiratory Syndrome Coronavirus-2. Cureus.

[12] Ellul M.A., Benjamin L., Singh B., Lant S., Michael B.D., Easton A., Kneen R., Defres S., Sejvar J., Solomon T. (2020). Neurological associations of COVID-19. Lancet Neurol..

[13] Lukiw W.J., Pogue A., Hill J.M. (2022). SARS-CoV-2 Infectivity and Neurological Targets in the Brain. Cell. Mol. Neurobiol..

[14] Taha M.J.J., Abuawwad M.T., Alrubasy W.A., Sameer S.K., Alsafi Y., Al-Busataji Y., Abu-Ismail L., Nashwan A.J. (2022). Ocular manifestations of recent viral pandemics: A literature review. Front. Med..

[15] Insausti-García A., Reche-Sainz J.A., Ruiz-Arranz C., Vázque Á.L., Ferro-Osuna M. (2022). Papillophlebitis in a COVID-19 patient Inflammation and hypercoagulable state. Eur. J. Ophthalmol..

[16] Azab M.A., Hasaneen S.F., Hanifa H., Azzam A.Y. (2021). Optic neuritis post-COVID-19 infection. A case report with meta-analysis. Interdisciplinary Neurosurgery. Adv. Techn. Case Manag..

[17] Makhija S.C., Walinjkar J.A., Sharma H.R., Morekar S.R., Natarajan S. (2020). Central retinal vein occlusion with COVID-19 infection as the presumptive etiology. Indian J. Ophthalmol..

[18] Romano D., Macerollo A., Giannaccare G., Mazzuca D., Borgia A., Romano V., Semeraro F., Ellis R. (2022). COVID-19 and Clinically Isolated Syndrome: Coincidence or Causative Link? A 12-Month Follow-Up Case Report. Appl. Sci..

[19] Raj A., Kaur N., Kaur N. (2021). Cavernous sinus thrombosis with central retinal artey occlusion in COVID-19: A case report and review of literature. Indian J. Ophthalmol..

[20] Lim T.H., Wai Y.Z., Chong J.C. (2021). Unilateral frosted branch angiitis in an human immunodeficiency virus-infected patient with concurrent COVID-19 infection: A case report. J. Med. Case Rep..

[21] Shiroma H.F., Lima L.H., Shiroma Y.B., Kanadani T.C., Nobrega M.J., Andrade G., de Moraes Filho M.N., Penha F.M. (2022). Retinal vascular occlusion in patients with the COVID-19 virus. Int. J. Retin. Vitr..

[22] Savino G., Gambini G., Scorcia G., Comi N., Fossataro C., Rizzo S. (2022). Orbital myositis and scleritis after anti-SARS-CoV-2 mRNA vaccines: A report of three cases. Eur. J. Ophthalmol..

[23] Franco S.V., Fonollosa A. (2022). IOptic Neuropathy After Administration of a SARS-CoV-2 Vaccine: A Report of 2 Cases. Am. J. Case Rep..

[24] Jin W., Tang Y., Wen C. (2021). An ocular adverse event in temporal association with COVID-19 vaccination in a patient with systemic lupus erythematosus: A case report. Hum. Vaccines Immunother..

[25] Subramony R., Lin L.C., Darren K., Knight D.K., Aminlari A., Belovarski I. (2021). Bilateral Retinal Detachments in a Healthy 22-year-old Woman After Moderna SARS-CoV-2 Vaccination. J. Emerg. Med..

[26] Tsuda K., Oishi A., Kitaoka T. (2022). Optic disc hemorrhage in a young female following mRNA coronavirus disease 2019 vaccination: A case report. J. Med. Case Rep..

[27] Camelia B.M., Iuliana A.A., Elena C.R., Giurgica M., Anca P., Grigoras C., Otilia O., Ionela N.D., Nicoleta A. (2021). Ophthalmoic Pathology Treated During COVID-19 at the Ophthalmology Clinic of “SF. Spiridon” County Clinical Emergency Hospital in Iasi, Romania. Med. Surg. J..

[28] Andronic D.G., Anton N., Niagu I.A., Bogdanici C.M. (2021). The incidence of pediatric eye injuries—Before and during the SARS-CoV-2 pandemic. Med. Surg. J. Rev. Med. Chir. Soc. Med. Nat. Iaşi.

